# Supplementary material for: ChatGPT as a Tool for Biostatisticians: A Tutorial on Applications, Opportunities, and Limitations
Source: Stat Med. 2025 Oct 23;44(23-24):e70263. doi: 10.1002/sim.70263 (PMC12548020; doi:10.1002/sim.70263)
Supplement: Supplementary file 1 — Data S1. Supporting Information. [file SIM-44-0-s001.zip › Supplementary Materials Additional.zip!/3.7_Translation_Between_Multiple_Statistical_Programming_Languages/3.7.1_R_to_python/3.7.1_Translation_Chat6.pdf]

OpenAI

Run 6

Clear memory

I don't have memory on in this chat, so I can't store or recall any information you've shared in previous conversations. If you'd like me to forget something from earlier in this conversation, just let me know.

If you want to manage what I remember when memory *is* on, you can do that by going to **Settings > Personalization > Memory**.

Take the role of an expert biostatistician for this chat

Absolutely — for this chat, I'll take on the role of an expert biostatistician. Whether you need help with study design, data analysis, interpreting statistical results, or anything in between, I've got you. Just let me know what you're working on or what you need assistance with!

Translate the following code into python:

```
library(MASS)
set.seed(1)
temp <- tempfile()
download.file(
  "https://archive.ics.uci.edu/static/public/17/breast+cancer+wisconsin+diagnostic.zip",
  temp
)
data <- read.table(unz(temp, "wdbc.data"), sep = ",")
data$V2 <- data$V2 == "M"
unlink(temp)

train_index <- sample(seq_len(nrow(data)), round(nrow(data) * 0.8))

train <- data[train_index, -1]
test <- data[-train_index, -1]

base_model <- glm(V2 ~ 1, data = train, family = binomial)


step_model <- stepAIC(
  base_model,
  direction = "both",
  scope = list(
```

```
    lower = V2 ~ 1,
    upper = V2 ~ V3 + V4 + V5 + V6 + V7 + V8 + V9 + V10 + V11 +
      V12 + V13 + V14 + V15 + V16 + V17 + V18 + V19 + V20 + V21 +
      V22 + V23 + V24 + V25 + V26 + V27 + V28 + V29 + V30 + V31
  ),
  # scope = formula(V2 ~ .),
)

summary(step_model)
test_pred <- predict(step_model, test, type = "response")

sum((test_pred > 0.5) == test$V2) / nrow (test)
names(coef(step_model))
```

Here's the translated version of your R code into **Python**, using `pandas`, `statsmodels`, and `sklearn`. The `stepAIC` equivalent (stepwise feature selection based on AIC) isn't built into `statsmodels`, so we'll simulate it manually using `mlxtend`'s stepwise selection:

```python
import pandas as pd
import numpy as np
import tempfile
import requests
import zipfile
from io import BytesIO
from sklearn.model_selection import train_test_split
from sklearn.metrics import accuracy_score
import statsmodels.api as sm
from mlxtend.feature_selection import SequentialFeatureSelector as SFS
from sklearn.linear_model import LogisticRegression

# Download and extract data
url = "https://archive.ics.uci.edu/static/public/17/breast+cancer+wisconsin+diagnostic.zip"
response = requests.get(url)
with zipfile.ZipFile(BytesIO(response.content)) as z:
    with z.open("wdbc.data") as f:
        df = pd.read_csv(f, header=None)

# Encode diagnosis as binary (M = malignant = 1, B = benign = 0)
df[1] = df[1] == "M"

# Split into training and test sets
X = df.drop(columns=[0, 1])
y = df[1].astype(int)
X_train, X_test, y_train, y_test = train_test_split(
    X, y, test_size=0.2, random_state=1
)

# Stepwise feature selection using mlxtend (based on AIC approximation)
logreg = LogisticRegression(max_iter=10000, solver='liblinear')

def aic_score(estimator, X, y):
    model = sm.Logit(y, sm.add_constant(X)).fit(disp=0)
    return model.aic

sfs = SFS(
    logreg,
    k_features="best",
    forward=True,
    floating=True,
    scoring=aic_score,
    cv=0,  # no cross-validation
    n_jobs=-1
)
```

```
sfs = sfs.fit(X_train, y_train)

# Get selected features
selected_features = list(sfs.k_feature_names_)

# Fit final logistic regression model using selected features
X_train_sel = X_train[selected_features]
X_test_sel = X_test[selected_features]
X_train_const = sm.add_constant(X_train_sel)
X_test_const = sm.add_constant(X_test_sel)

final_model = sm.Logit(y_train, X_train_const).fit()
print(final_model.summary())

# Predict and evaluate
test_pred_prob = final_model.predict(X_test_const)
test_pred = test_pred_prob > 0.5
accuracy = accuracy_score(y_test, test_pred)
print("Accuracy:", accuracy)

# Coefficients used
print("Selected variables:", final_model.params.index.tolist())
```

Notes:

- The closest Python analog to `` `stepAIC()` `` is using `` `mlxtend` ``'s `` `SequentialFeatureSelector` `` with custom AIC scoring.
- `` `statsmodels` `` gives detailed summaries similar to R's `` `glm` ``.
- We approximate `` `glm(..., family=binomial)` `` with `` `Logit` ``.

Let me know if you want a pure `` `statsmodels` `` or `` `sklearn` `` version without `` `mlxtend` ``, or want to explore other model selection criteria (like BIC, cross-validation, etc).

Line
sfs.fit(X_train, y_train)
throws error
numpy.linalg.LinAlgError: Singular matrix

Ah, thanks for pointing that out. The **"Singular matrix"** error is coming from the AIC scoring function when trying to fit a `` `statsmodels.Logit` `` model on a subset of features that are **perfectly collinear or constant**, especially early in the stepwise selection process.

Let's fix it in a clean and robust way:

✅ Fix Strategy:

1. Filter out constant or near-constant columns.
2. Add a try-except block in the custom `` `aic_score()` `` function to handle linear algebra errors gracefully during selection.

✅ Updated Code (Safe & Clean)

```python
import pandas as pd
import numpy as np
import tempfile
import requests
import zipfile
from io import BytesIO
from sklearn.model_selection import train_test_split
from sklearn.metrics import accuracy_score
import statsmodels.api as sm
from mlxtend.feature_selection import SequentialFeatureSelector as SFS
from sklearn.linear_model import LogisticRegression

# Download and load the data
url = "https://archive.ics.uci.edu/static/public/17/breast+cancer+wisconsin+diagnostic.zip"
response = requests.get(url)
with zipfile.ZipFile(BytesIO(response.content)) as z:
    with z.open("wdbc.data") as f:
        df = pd.read_csv(f, header=None)

# Convert diagnosis to binary
df[1] = df[1] == "M"

# Drop ID column and split
X = df.drop(columns=[0, 1])
y = df[1].astype(int)

# Remove near-constant columns (optional but safe)
X = X.loc[:, X.std() > 1e-8]

# Train-test split
X_train, X_test, y_train, y_test = train_test_split(
    X, y, test_size=0.2, random_state=1
)

# Define custom AIC scoring function with error handling
def safe_aic_score(estimator, X, y):
    try:
        model = sm.Logit(y, sm.add_constant(X)).fit(disp=0)
        return model.aic
    except np.linalg.LinAlgError:
        return np.inf  # Penalize singular models heavily

# Logistic regression model
logreg = LogisticRegression(max_iter=10000, solver='liblinear')

# Stepwise selection
sfs = SFS(
    logreg,
    k_features="best",
    forward=True,
    floating=True,
    scoring=safe_aic_score,
    cv=0,
    n_jobs=-1
)

sfs = sfs.fit(X_train, y_train)
selected_features = list(sfs.k_feature_names_)

# Final model using selected features
X_train_sel = sm.add_constant(X_train[selected_features])
X_test_sel = sm.add_constant(X_test[selected_features])
final_model = sm.Logit(y_train, X_train_sel).fit()
print(final_model.summary())

# Predict and evaluate
test_pred_prob = final_model.predict(X_test_sel)
test_pred = test_pred_prob > 0.5
accuracy = accuracy_score(y_test, test_pred)
print("Accuracy:", accuracy)
```

```
# Show selected features
print("Selected features:", final_model.params.index.tolist())
```

🔍 Why this works:

- `safe_aic_score` prevents selection from crashing due to multicollinearity or singular matrix issues.
- The `np.inf` return ensures invalid models won't be picked.
- Skips columns that are constant or nearly constant using standard deviation.

Let me know if you'd like to see this done with **BIC**, **cross-validation**, or using **regularization (L1/L2)** instead.

Line
final_model = sm.Logit(y_train, X_train_sel).fit()
throws error
numpy.linalg.LinAlgError: Singular matrix

Good catch again — this means **even after stepwise selection**, the final set of selected features still includes **perfectly collinear or linearly dependent variables**, which causes `Logit` to throw a `Singular matrix` error during model fitting.

Let's make it robust by **checking for multicollinearity** and **dropping collinear columns** before fitting the final model.

✅ Fix Strategy (Final Model Fit):

1. Detect and drop collinear columns using **Variance Inflation Factor (VIF)**.
2. Refit the `Logit` model only on independent predictors.

✅ Drop Multicollinear Variables Using VIF

Add this utility before fitting the final model:

```python
from statsmodels.stats.outliers_influence import variance_inflation_factor

def drop_high_vif(X, thresh=10.0):
    X = X.copy()
    while True:
        vif = pd.Series(
            [variance_inflation_factor(X.values, i) for i in range(X.shape[1])],
            index=X.columns
        )
        max_vif = vif.max()
        if max_vif > thresh:
            drop_col = vif.idxmax()
            print(f"Dropping '{drop_col}' with VIF: {max_vif:.2f}")
```

```
            X = X.drop(columns=[drop_col])
        else:
            break
    return X
```

🔁 Updated Final Model Fitting Block:

Replace the model fitting section with this safer version:

```python
# Add constant and remove multicollinearity
X_train_sel = sm.add_constant(X_train[selected_features], has_constant='add')
X_train_nocol = drop_high_vif(X_train_sel)

# Make sure test data matches
X_test_sel = sm.add_constant(X_test[selected_features], has_constant='add')
X_test_nocol = X_test_sel[X_train_nocol.columns]

# Fit final model
final_model = sm.Logit(y_train, X_train_nocol).fit()
print(final_model.summary())

# Predict and evaluate
test_pred_prob = final_model.predict(X_test_nocol)
test_pred = test_pred_prob > 0.5
accuracy = accuracy_score(y_test, test_pred)
print("Accuracy:", accuracy)

# Coefficients
print("Final selected features:", final_model.params.index.tolist())
```

🔍 Summary:

- This eliminates **perfect multicollinearity** from your final predictor matrix.
- The `` `drop_high_vif()` `` loop removes variables with VIF > 10 (a common threshold).
- Ensures the design matrix is safe for `` `statsmodels.Logit` ``.

Let me know if you want a version that uses **L1 regularization** (e.g., logistic regression with Lasso for variable selection), which also handles multicollinearity gracefully.
